# Experimental Warming Has Not Affected the Changes in Soil Organic Carbon During the Growing Season in an Alpine Meadow Ecosystem on the Qinghai–Tibet Plateau

**DOI:** 10.3389/fpls.2022.847680

**Published:** 2022-03-18

**Authors:** Yue Yang, Guoxi Shi, Yongjun Liu, Li Ma, Zhonghua Zhang, Shengjing Jiang, Jianbin Pan, Qi Zhang, Buqing Yao, Huakun Zhou, Huyuan Feng

**Affiliations:** ^1^Ministry of Education Key Laboratory of Cell Activities and Stress Adaptations, School of Life Sciences, Lanzhou University, Lanzhou, China; ^2^Key Laboratory of Utilization of Agriculture Solid Waste Resources in Gansu Province, College of Bioengineering and Biotechnology, Tianshui Normal University, Tianshui, China; ^3^Key Laboratory of Restoration Ecology of Cold Area in Qinghai Province, Northwest Institute of Plateau Biology, Chinese Academy of Sciences, Xining, China; ^4^State Key Laboratory of Grassland and Agro-Ecosystems, College of Pastoral Agriculture Science and Technology, Lanzhou University, Lanzhou, China

**Keywords:** climate warming, seasonal differences, dynamic of soil organic carbon, soil microbiota, the growing season

## Abstract

The effects of climate warming and season on soil organic carbon (SOC) have received widespread attention, but how climate warming affects the seasonal changes of SOC remains unclear. Here, we established a gradient warming experiment to investigate plant attributes and soil physicochemical and microbial properties that were potentially associated with changes in SOC at the beginning (May) and end (August) of the growing season in an alpine meadow ecosystem on the Qinghai–Tibet Plateau. The SOC of August was lower than that of May, and the storage of SOC in August decreased by an average of 18.53 million grams of carbon per hectare. Warming not only failed to alter the content of SOC regardless of the season but also did not affect the change in SOC during the growing season. Among all the variables measured, microbial biomass carbon was highly coupled to the change in SOC. These findings indicate that alpine meadow soil is a source of carbon during the growing season, but climate warming has no significant impact on it. This study highlights that in the regulation of carbon source or pool in alpine meadow ecosystem, more attention should be paid to changes in SOC during the growing season, rather than climate warming.

## Introduction

Since the 1850s, the global surface temperature has increased by approximately 1.09°C (IPCC, 2021). Climate warming is one of the detrimental aspects of climate change (Lippmann et al., 2019), and it has a strong impact on ecosystem processes (Singh et al., 2010; Scheffers et al., 2016). Numerous studies have focused on the effects of climate warming on the aboveground ecosystem (Klein et al., 2004; Post and Pedersen, 2008; Elmendorf et al., 2012; Zhang et al., 2017). However, compared with the key role of belowground part of an ecosystem in the regulation of material and energy flows between the aboveground and belowground parts of an ecosystem (Peng et al., 2021; Zhang et al., 2021), research on the impacts of climate warming on the belowground part of an ecosystem is still lacking. This greatly limits the accuracy of predictions of the ecological consequences of climate warming.

As the largest repository of organic carbon in the world, the soil stores approximately 2 trillion tons of carbon (Hutchins et al., 2019). This is approximately twice the carbon storage of the atmosphere and three times that of terrestrial plants (Lal, 2004; Schmidt et al., 2011). The annual carbon flux out of the soil is as high as 6–8 billion tons, which is 6- to 8-fold higher than the carbon emissions produced by human activities (Schlesinger and Andrews, 2000; Bond-Lamberty and Thomson, 2010). Thus, any slight change in the soil carbon pool can lead to an increase in the concentration of carbon dioxide in the atmosphere, which will further accelerate climate warming *via* positive feedback (García-Palacios et al., 2021; Zhu et al., 2021). In view of the important regulatory role of the soil carbon pool in the global carbon cycle, numerous evidence has shown that experimental warming has demonstrated negative (Feng et al., 2017; Wang et al., 2019), neutral (Zhang et al., 2016; Jia et al., 2017; Guan et al., 2018; Ding et al., 2019; Wang et al., 2019; Tian et al., 2021), and positive effects on the content of soil organic carbon (SOC; Xu et al., 2015; Zheng et al., 2020; Tian et al., 2021). Such controversy indicates that the responses of the content of SOC to warming are complex and merit further investigation (Zhang et al., 2020; Craig et al., 2021).

Although the responses of content of SOC to warming have been widely documented, the mechanisms of how elevated temperatures impact SOC remain unclear. The soil carbon cycle model is included in the Earth System Models, which will be used to predict the turnover of SOC (Campbell and Paustian, 2015; Zhang et al., 2020; Fan et al., 2021). In one aspect of this model, warming will affect plant growth by changing the availability of soil nutrients, thereby affecting the input of plants to the SOC pool; alternatively, the temperature increase caused by the warming treatment will accelerate the loss of SOC by changing the metabolism of soil microorganisms (Luo et al., 2001; Luo, 2007; Conant et al., 2011; Campbell and Paustian, 2015). These potential pathways indicate that the effect of elevated temperatures on the content of SOC can approximately be classified into direct, that is, they affect soil microbial metabolism, or indirect, that is, they change the plant biomass or soil properties. Therefore, there is still an urgent need for a more systematic study to determine the potential impact of classified temperature on SOC and its underlying mechanism.

The Qinghai–Tibet Plateau, with an average altitude that exceeds 4,000 m, is known as “the roof of the world” and has experienced striking warming at the rate of 0.4°C per decade over the past 50 years, which is double that of the global mean (Gao et al., 2014; Fu et al., 2021). The dominant ecosystem of the region is composed of alpine meadows (Wang et al., 2014). The combination of alpine meadows and alpine steppes contributes 2.32 billion tons of carbon (69.3%) to the soil total organic carbon stock on the Qinghai–Tibetan Plateau, and alpine meadows had the highest content of carbon in this region (Wang et al., 2002; Guan et al., 2018). Since the SOC in high-altitude regions is extremely sensitive to climate warming (García-Palacios et al., 2021), alpine meadows are ideal places to study the response of terrestrial ecosystems to climate warming (Zhang et al., 2016). Currently, a large number of studies have been conducted to examine the impact of climate warming (Xu et al., 2015; Jia et al., 2017; Ding et al., 2019; Chen et al., 2020) or seasons (Luo and Weng, 2011) on the content of SOC. Nevertheless, to our knowledge, few studies have focused on changes in the content of SOC between the beginning and the end of the growing season along a temperature gradient in an alpine meadow ecosystem. This substantially limits our understanding of the driving mechanism of changes in SOC during the growth season under the warming scenario because both plant and soil microbial communities in alpine meadow ecosystems are highly vital during the growing season, and the effects of warming on SOC are always coupled with seasonal changes. Therefore, to explore the impact of climate warming on the changes of organic carbon during the growing season, we conducted an 8-year warming gradient experiment with open-top chambers (OTCs) to reveal the impact of experimental warming on the differences of SOC between the beginning (May) and end (August) of the growing season. Previous studies have confirmed that warming can facilitate plant growth and increase soil microbial activity and soil respiration (Conant et al., 2011; Luo and Weng, 2011). Thus, we hypothesized that warming could facilitate the loss of SOC regardless of the season (H1). In addition, since the end of the growing season has a higher soil temperature than the beginning of the growing season in alpine meadow ecosystems, we also predicted that the content of SOC at the end of the growing season would be lower than that at the beginning of the growing season (H2). Moreover, warming can reduce the differences in soil temperature between the beginning and end of the growing season by prolonging the growing season (Luo and Weng, 2011). We also hypothesized that as the temperature increased, the seasonal difference of SOC would gradually decrease (H3).

## Materials and Methods

### Field Site Description and Experimental Setup

This study was conducted at the Haibei Research Station of the Chinese Academy of Sciences (37°29′N, 101°12′E; 3,220 ma.s.l.), China. This region has a typical continental semi-humid plateau climate with long cold dry winters and short warm wet summers. The average annual temperature is −1.7°C and ranges from −15.2°C in January to 9.9°C in July. The average annual precipitation is 561 mm, and more than 80% of the annual precipitation is concentrated during the growing season. The growing season in this region is from May to August. The vegetation of this region is dominated by graminoids and forbs, such as *Kobresia humilis* Sergiev., *Stipa aliena* Keng., *Elymus nutans* Griseb., *Poa pratensis* L., *Oxytropis ochrocephala* Bunge., *Medicago ruthenica* (L.) Trautv., and *Gentiana straminea* Maxim.

The field experiment was established in July 2011 in a fenced 50 × 50 m^2^ flat area that was previously used as a winter pasture to examine how the alpine meadow ecosystem responds to gradient warming. Twenty-five plots composed of five temperature treatments (ambient temperature and four warming treatments) with five repetitions were distributed into subplots divided into five rows and five columns based on a random block design (Supplementary Figure S1A). These four warming treatments were achieved with open-top chambers that were designed as four types of sample rounds from large to small, corresponding to the magnitude of warming from weak to strong (Supplementary Figure S1A). These OTCs were constructed with 1 mm thick fiberglass and were 40 cm high. The diameters of the bottom/top of the chambers were 2.05 m/1.60 m for W1, 1.75 m/1.30 m for W2, 1.45 m/1.00 m for W3, and 1.15 m/0.70 m for W4. During the experiment, all the open-top chambers were maintained at the plots throughout the year (Supplementary Figure S1). Decagon ECT sensors (Decagon Devices, Pullman, WA, United States) were used to record the daily air temperature at 20 cm high and 10 cm aboveground and soil temperature at a depth of 15 cm belowground. After monitoring, these four types of OTC treatments (W1–W4) successfully produced increased soil temperature gradients. The mean increase in soil temperature at the beginning of growing season (May) was 0.73°C, 1.60°C, 1.73°C, and 2.25°C in W1 to W4, respectively, while the mean increase in soil temperature at the end of growing season (August) was 0.52°C, 0.97°C, 1.49°C, and 1.57°C, respectively (Supplementary Table S1). Compared with the beginning of the growing season (May), the soil temperature at the end of the growing season (August) increased by 5.82°C (W0), 5.61°C (W1), 5.19°C (W2), 5.58°C (W3) and 5.14°C (W4; Supplementary Table S1).

### Soil Sampling and the Plant Community Survey

Soil samples were collected at the beginning (on May 3) and end of the growing season (August 28) in 2019, respectively. Three soil cores (5.0 cm in diameter and 25 cm deep) were randomly collected in each plot and homogenized into one composite sample (2 seasons × 5 treatments × 5 repetitions = 50 soil samples). Each soil composite sample was then divided into two subsamples. One was used to extract DNA, and the other was used to determine the soil physicochemical and microbial properties. All the soil samples were stored at 4°C during the sampling process (within 48 h) and were transferred into −20°C storage once they had been transported to the laboratory. The plant community survey was only performed at the end of growing season because it was difficult to identify plant species when the plant community had just turned green at the beginning of growing season. The plant community in each plot was surveyed using a 50 × 50 cm^2^ sample quadrat. All the shoots in each quadrat were cut at the ground surface and grouped by plant species. The shoots of each species were then weighed after drying at 80°C for 48 h, and aboveground biomass of plant community was calculated as sum of shoot biomass of each species.

### Analysis of Soil Properties

The soil moisture was measured gravimetrically after drying at 105°C for 12 h. The soil pH was measured with a pH electrode (Sartorius PB-10; Sartorius AG, Göttingen, Germany). SOC, dissolved organic carbon (DOC), and nitrogen were measured using an Elementar analysis system (CHNS; Elementar Analysensysteme GmbH, Langenselbold, Germany; Cabrera and Beare, 1993; Jones and Willett, 2006; Makarov et al., 2013). Both soil available nitrogen (as ammonia and nitrate) and available phosphorus were measured using FIAstar analyzer 5,000 (FOSS, Hillerød, Denmark). The rate of soil respiration for each warming treatment was measured 10 days per month with an LI-8100A Automated Soil CO_2_ Flux System (LICOR, Inc., Lincoln, NE, United States) throughout the growing season (from May to August; Ren et al., 2021). SOC storage was calculated based on the SOC, bulk density, and soil sampling depth (Emde et al., 2021). Both microbial biomass carbon (MBC) and microbial biomass nitrogen (MBN) were measured using the chloroform fumigation direct extraction method (Brookes et al., 1985; Vance et al., 1987). The activities of four soil extracellular enzymes that decompose SOC, including the labile carbon decomposing enzymes amylase and sucrase and the recalcitrant carbon decomposing enzymes cellulase and peroxidase, were assayed as described by Xun et al. (2018), Yin et al. (2014), and Jiang et al. (2021).

### Soil DNA Extraction, Real-Time Quantitative PCR, and Amplicon Sequencing

Total DNA was extracted from 0.5 g of each soil sample using an E.Z.N.A.® Soil DNA Kit (Omega Bio-tek, Norcross, GA, United States) following the manufacturer’s instructions. The primer pair combination of ITS1F (5'-CTTGGTCATTTAGAGGAAGTAA-3')-ITS2R (5'-GCTGCGTTCTTCATCGATGC-3') was then used to determine the number of gene copies of fungal DNA (ITS), while the primer pair combination of 338F (5'-ACTCCTACGGGAGGCAGCAG-3')-806R (5'-GGACTACHVGGGTWTCTAAT-3') were used to determine the number of gene copies of bacteria (16S rRNA) using a QuantStudio™ 6 Flex Real-Time PCR System (Applied Biosystems, Waltham, MA, United States; Majorbio Bio-Pharm Technology Co., Ltd., Shanghai, China). The target fragment of the primer combination ITS1F-ITS2R was ~300 bp that targeted the ITS1 region (White et al., 1900), while the target fragment of the primer combination 338F-806R was ~468 bp in the V3-V4 region (Mori et al., 2014). The primers used for high-throughput sequencing of fungi (ITS) and bacteria (16S rRNA) were the same as the primers for qPCR. The amplification with primer pair ITS1F-ITS2R was conducted in a 20 μl reaction that consisted of 10 ng for the total DNA template, 0.8 μl of each primer (5 μM), 2 μl 10 × TransStart FastPfu buffer, 2 μl dNTPs (2.5 mM), and 0.2 μl TaKaRa rTaq DNA Polymerase. The thermal cycling conditions were as follows: 95°C for 3 min, 27 cycles of 95°C for 30 s, 55°C for 30 s 72°C for 45 s, and 72°C for 10 min. The amplification with primer pair 338F-806R was similar to those of the ITS1F-ITS2R amplifications with the exception of the use of 0.4 μl TransStart FastPfu DNA Polymerase. The PCR reactions were performed in triplicate. The sizes of both fungal and bacterial amplicons were confirmed separately using 2% agarose gel electrophoresis, purified using an AxyPrep DNA Gel Extraction Kit (Axygen Biosciences, Union City, CA, United States), and quantified using a Quantus™ Fluorometer (Promega, Madison, WI, United States). Purified amplicons were pooled in equimolar amounts and paired-end sequenced on an Illumina MiSeq PE300 platform (Illumina, San Diego, CA, United States) by Majorbio Bio-Pharm Technology Co., Ltd. (Shanghai, China) using a NEXTFLEX® Rapid DNA-Seq Kit (Bioo Scientific Corporation, Austin, TX, United States) for library construction. All the raw sequencing reads were quality-filtered by FASTP (ver. 0.20.0; Chen et al., 2018) and merged by FLASH (ver. 1.2.7; Magoc and Salzberg, 2011). Operational taxonomic units (OTUs) with a 97% similarity cutoff (Stackebrandt and Goebel, 1994; Edgar, 2013) were clustered using UPARSE (ver. 7.1; Edgar, 2013). The taxonomy of each representative OTU sequence was analyzed using RDP Classifier (ver. 2.2; Wang et al., 2007) against the ITS database (UNITE ver. 7,^1^ for fungal OTUs) and the 16S rRNA database (Silva ver. 138,^2^ for bacterial OTUs) using a confidence threshold of 0.7. The sequence raw datasets in this study were deposited in the NCBI Sequence Read Archive (SRA) with accession numbers: PRJNA719774 (ITS-BioProject) and PRJNA719776 (16S rRNA-BioProject).

### Statistical Analyses

The statistical analyses were primarily conducted using R 4.0.5.^3^ A linear mixed-effects (LME) model (“lme” model) was applied to analyze the individual effects of warming and season on soil, plant, and microbial variables using the “nlme” and “nlme” functions in R (Pinheiro et al., 2019), in which both warming and season were treated as fixed effects and block as a random effect. Significant differences between treatments were tested using the *post-hoc* multiple comparisons Tukey method at a 95% significance level (*p* ≤ 0.05). A rarefaction curve of microbial species richness was estimated using the “rarecurve” function of the “vegan” package in R (Oksanen et al., 2019). A taxonomy Circlize of microbial community composition at the phylum level was summarized using the “tax_circlize” function of the “amplicon” package in R.^4^ Dissimilarities of the plant or microbial community were depicted with principal coordinate analysis (PCoA) ordination based on the Bray–Curtis dissimilarity index using the “cmdscale” function of the “stats” package in R (Oksanen et al., 2007). A permutational multivariate analysis of variance (PERMANOVA) was used to determine the effects of warming or season on both plant and microbial communities using the “adonis” function of the “vegan” package in R (Oksanen et al., 2019). Linear discriminant analysis (LDA) effect size (LEfSe ver. 1.0)^5^ was performed to assess significant differences of the fungal and bacterial taxa from Kingdom; Domain, not Kingdom, was used as the basic classification level for bacteria to genus levels (Segata et al., 2011).

The natural logarithm ratio was used to interpret the rate of change of all the variables measured during the growing season. For example, the rate of change of SOC during the growing season was calculated as $\Delta {SOC}_{\mathrm{content}\;\left(\%\right)}=\left(e^{\ln\left(\frac{{SOC}_{Aug}}{{SOC}_{\mathrm{May}}}\right)}-1\right)\times 100$, where SOC_Aug_ and SOC_May_ represent the content of SOC in August and May, respectively (Emde et al., 2021). Considering that the score values of the PCoA axis of the plant or microbial communities cannot be characterized by the natural logarithm owing to a negative value, we applied the ratio of γ-diversity to α-diversity to characterize the rate of change of the composition of plant or microbial community during the growing season using the “diversity” function of the “vegan” package in R (Oksanen et al., 2019).

Linear regression analysis was performed to assess the relationships between the content of SOC or the rates of change of SOC with all plant, soil, and microbial variables using the linear mixed-effects regression with block as a random effect. A random forest test was performed to predict response variables that are significantly related to the content of SOC (or the rate of change of SOC) using the “rfPermute” function of the “rfPermute” package in R (Archer, 2013). A structural equation model (SEM) was also constructed using SPSS AMOS 21.0 (IBM, Inc., Armonk, NY, United States).

## Results

### Soil Properties and Plant Community

**Figure 1:**
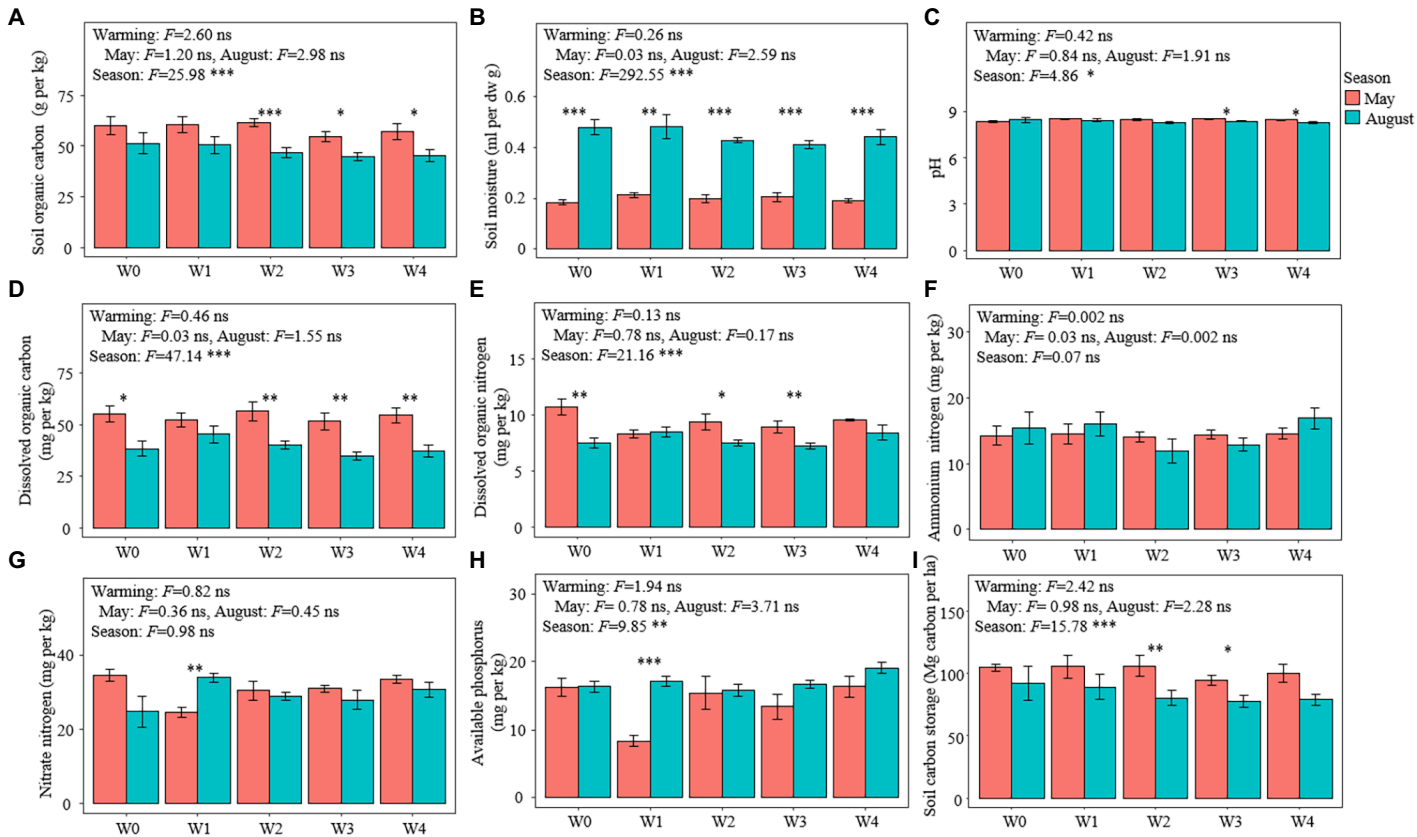
Soil characteristics in the different treatments. **(A)** Soil organic carbon content, **(B)** soil moisture, **(C)** pH, **(D)** dissolved organic carbon content, **(E)** dissolved organic nitrogen content, **(F)** ammonium nitrogen content, **(G)** nitrate nitrogen content, **(H)** available phosphorus content, and **(I)** soil carbon storage. Bars represent the means ± SE (*n* = 5). Significant differences among the treatments were tested using Tukey’s honest significant difference test (*p* ≤ 0.05). The *F*-values and significance shown correspond to the linear mixed-effects model. ^*^p < 0.05, ^**^p < 0.01, ^***^p < 0.001. SE, standard error.

Warming had no significant effect on all the soil variables measured, but the season had significant effects on other soil physical and chemical properties except for soil ammonium nitrogen and nitrate nitrogen (Figure 1). The soil moisture and available phosphorus in May were lower than those in August, while the contents of SOC, DOC, dissolved organic nitrogen, and soil carbon storage in May were higher than those in August (Figure 1). Moreover, warming significantly reduced the aboveground biomass and species richness of the plant communities but did not affect the species composition of these communities (Supplementary Figure S2).

### Soil Microbial Biomass, Soil Respiration Rate, and Soil Extracellular Enzyme Activity

**Figure 2:**
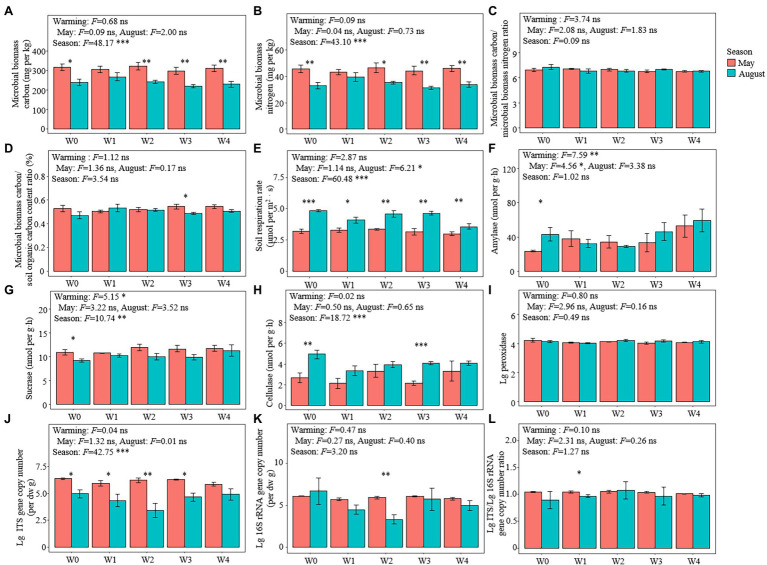
Microbial properties in the different treatments. **(A)** microbial biomass carbon (MBC) content, **(B)** microbial biomass nitrogen (MBN) content, **(C)** MBC/MBN ratio, **(D)** MBC/SOC ratio (%), **(E)** soil respiration rate, **(F)** amylase activities, **(G)** sucrase activities, **(H)** cellulase activities, **(I)** lg_10_ transformed peroxidase activities (lg peroxidase), gene copy numbers of **(J)** eukaryotic (lg ITS) and **(K)** bacteria (lg 16S rRNA), and **(L)** lg ITS/lg 16S rRNA ratio. Bars represent the means ± SE (*n* = 5). Significant differences among the treatments were tested using Tukey’s honest significant difference test (*p* ≤ 0.05). The *F*-values and significance shown correspond to the linear mixed-effects model. ^*^p < 0.05, ^**^p < 0.01, ^***^p < 0.001. SE, standard error.

Except for W1, both the MBC and MBN of May were higher than those of August (Figures 2A,B). There was no significant difference in the ratio of MBC/MBN and MBC/SOC among the treatments (Figures 2C,D). The rates of soil respiration in May were lower than those in August under all the warming treatments (Figure 2E). The activity of amylase in May was lower than that in August, and the activity of sucrase in May was higher than that in August under the W0 treatment (Figures 2F,G). The activity of cellulase in both W0 and W3 in May was lower than that in August, while there were no significant differences in the activity of peroxidase between May and August under all the warming treatments (Figures 2H,I).

### Microbial Community Diversity

Warming had no significant effect on the fungal (ITS) gene copy number (Figure 2J), bacterial gene copy number (Figure 2K), and the ratio of fungal/bacterial gene copy number (Figure 2L), while seasons significantly affected the gene copy number of fungi (Figure 2J). The gene copy number of fungi in May under all the warming treatments (except for W4) was higher than that in August (Figure 2J). Rarefaction curves showed that the high-throughput sequencing effectively identified the species richness of both fungal and bacterial communities (Supplementary Figure S3). A total of 307 genera of 36 classes of fungi were detected in 13 phyla, and 407 genera of 81 classes of bacteria were detected in 36 phyla (Supplementary Table S2). Ascomycetes were the dominant fungal taxon, comprising 65.27% of the total number of reads, while Proteobacteria was the dominant taxon of bacteria, comprising 30.90% of the total number of bacterial reads (Supplementary Figure S4).

**Figure 3:**
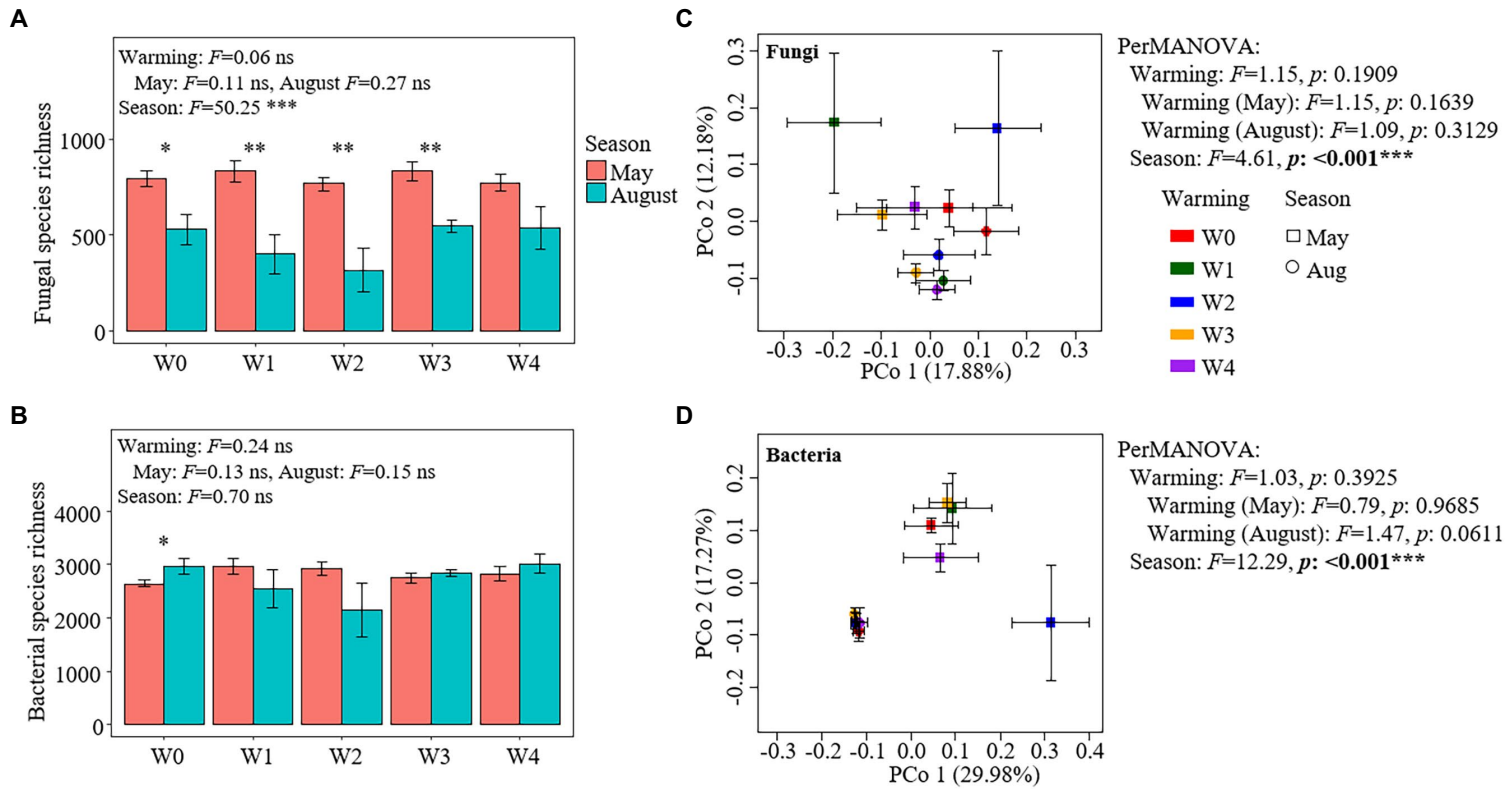
Species richness and the results of principal coordinate analysis (PCoA) and permutational multivariate analysis of variance of fungal **(A,C)** and bacterial communities **(B,D)**.

Warming had no significant effect on the species richness of fungal and bacterial communities (Figures 3A,B). The season had a significant effect on the richness of fungal species but had no significant effect on the richness of bacterial species (Figures 3A,B). The richness of fungal species was higher in May than that in August (except for W4) under all the warming treatments, while the richness of bacterial species in May was significantly lower than that in August under the W0 treatment (Figures 3A,B). In addition, warming had no significant effect on the taxonomic composition of fungal and bacterial communities, while season had a significant effect on them (Figures 3C,D). The relative abundances of 21 fungal OTUs and 310 bacterial OTUs were higher in May than in August, while the relative abundances of only four bacterial OTUs were lower in May than in August (Supplementary Figure S5).

### Relationships of Plant Attributes, Soil Properties, and Microbial Properties With the Content of SOC

**Figure 4:**
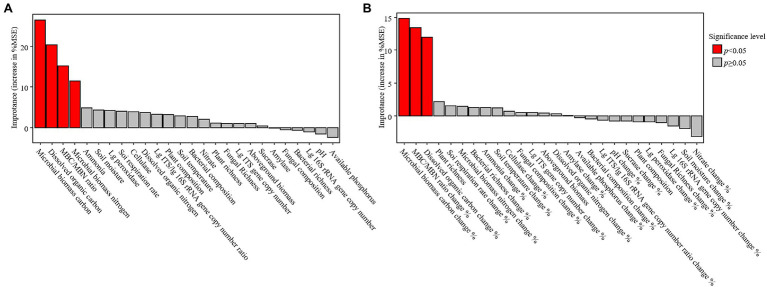
Results of the random forest analysis for soil organic carbon **(A)** and the changes in soil organic carbon during the growing season **(B)**. Variables that significantly correlated with changes of the independent variable are represented by red bars. MSE, mean square error. Lg, log_10_ transformed.

**Figure 5:**
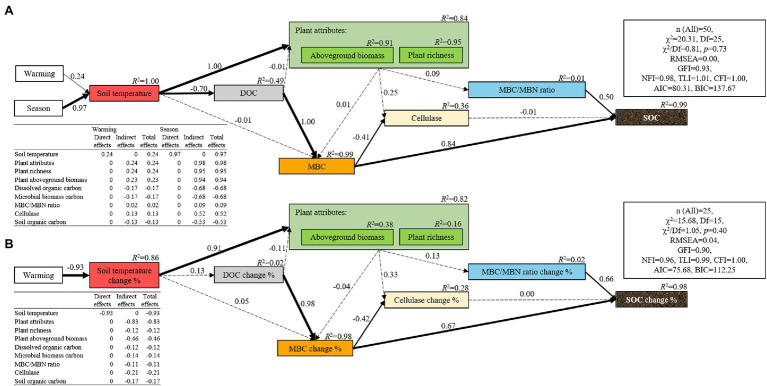
Structural equation model for soil organic carbon **(A)** and the change of soil organic carbon during the growing season **(B)**. The thickness of the arrows indicates the strength of the effect, and the number along each arrow represents the coefficient of the path. The dotted lines indicate paths that are not significant. The *R*^2^ value represents the proportion of variance that each variable can explain.

Soil organic carbon positively correlated with the DOC, MBC, MBN, and sucrase activity but negatively correlated with the soil temperature, aboveground biomass, rate of soil respiration, and cellulase activity (Table 1). The random forest analysis confirmed that MBC, DOC, MBC/MBN, and microbial biomass nitrogen were response variables that highly correlated with changes in the SOC (Figure 4A). The structural equation models also showed that the season, but not warming, mediated changes in the SOC through MBC (Figure 5A).

**Table 1:** Relationships between soil organic carbon content and plant attributes and soil and microbial properties.

Variable	Slope	*F*	*p*-Adjusted
*Plant attributes*			
Aboveground biomass	−0.02	19.79	**0.0003** ^***^
Plant richness	−0.47	18.80	**0.0003** ^***^
Plant composition	−12.77	28.26	**<0.0001** ^***^
*Soil properties*			
Soil temperature	−2.08	30.57	**<0.0001** ^***^
Soil moisture	−35.02	16.34	**0.0005** ^***^
pH	13.04	2.12	0.1799
Dissolved organic carbon	0.72	90.21	**<0.0001** ^***^
Dissolved organic nitrogen	4.20	31.21	**<0.0001** ^***^
Ammonia	0.41	1.02	0.3599
Nitrate	0.08	0.10	0.7536
Available phosphorus	−0.57	2.68	0.1404
*Microbial properties*			
Soi respiration rate	−5.32	11.27	**0.003** ^**^
Microbial biomass carbon (MBC)	0.16	129.15	**<0.0001** ^***^
Microbial biomass nitrogen (MBN)	0.83	40.09	**<0.0001** ^***^
MBC/MBN ratio	12.27	16.54	**0.0005** ^**^
Lg ITS gene copy number	3.65	14.00	**0.001** ^**^
Lg 16S rRNA gene copy number	0.40	0.26	0.6603
Lg ITS/Lg 16S rRNA gene copy number	12.98	3.50	0.0974
Fungal richness	0.02	10.90	**0.0031** ^**^
Bacterial richness	0.00	3.11	0.1151
Fungal composition	−12.98	14.00	**0.001** ^**^
Bacterial composition	−22.52	9.14	**0.0065** ^**^
Amylase activity	−0.03	0.15	0.7296
Sucrase activity	1.91	5.11	**0.0434** ^*^
Cellulase activity	−3.60	15.98	**0.0005** ^***^
Peroxidase activity	12.98	2.23	0.1754

Values of *p* correspond to the linear mixed-effects model regression. Significant correlations (*p* < 0.05) are formatted in bold.

* *p* < 0.05;

** *p* < 0.01;

*** *p* < 0.001.

### Relationships of the Change in Plant Attributes and Soil and Microbial Properties With the Change in Content of SOC During the Growing Season

The rate of change of soil and microbial properties were not significantly different among the warming treatments (Supplementary Figure S6). The rates of change of the SOC were positively related to the rates of seasonal change of DOC, MBC, and MBC/MBN (Table 2). Although warming had no significant effect on the rates of change of SOC, the SOC decreased significantly during the growing season only under the warming treatments (W2, W3, and W4; Supplementary Figure S6). The random forest analysis also confirmed that the rates of change of MBC, MBC/MBN, and DOC were variables that were associated with the change of SOC during the growing season (Figure 4B). The structural equation model that was conducted to measure the causal effect of warming on the change of SOC had also shown that warming failed to alter the change of SOC by the seasonal changes in soil temperature, but the change of MBC had a significant effect on it (Figure 5B).

**Table 2:** Relationships between the change of soil organic carbon with the change of plant attributes and soil and microbial properties during the growing season.

Variable	Slope	*F*	*p*-Adjusted
*Plant attributes*			
Aboveground biomass	−0.01	0.12	0.8485
Plant richness	0.07	0.01	0.9403
Plant composition	−26.32	0.53	0.7806
*Soil properties*			
Soil temperature change %	0.38	1.06	0.6784
Soil moisture change %	−0.02	0.06	0.8485
pH change %	1.72	0.75	0.7806
Dissolved organic carbon change %	0.81	23.48	**0.0009** ^***^
Dissolved organic nitrogen change %	0.52	6.31	0.1128
Ammonia change %	0.19	2.10	0.4640
Nitrate change %	−0.08	0.32	0.8272
Available phosphorus change %	0.05	0.42	0.7971
*Microbial properties*			
Soi respiration rate change %	−0.10	0.28	0.8272
Microbial biomass carbon (MBC) change %	1.06	39.39	**<0.0001** ^***^
Microbial biomass nitrogen (MBN) change %	0.55	5.01	0.1595
MBC/MBN ratio change %	1.70	33.90	**<0.0001** ^***^
Lg ITS gene copy number change %	0.24	1.70	0.5340
Lg 16S rRNA gene copy number change %	−0.03	0.09	0.8485
Lg ITS/Lg 16S rRNA gene copy number change %	0.17	1.20	0.6736
Fungal richness change %	0.07	0.21	0.8477
Bacterial richness change %	0.24	2.73	0.3637
Fungal composition change %	−0.11	0.51	0.7806
Bacterial composition change %	−0.09	0.60	0.7806
Amylase activity change %	−0.01	0.06	0.8485
Sucrase activity change %	0.06	0.08	0.8485
Cellulase activity change %	−0.10	6.16	0.1128
Peroxidase activity change %	1.25	3.01	0.3577

Values of *p* shown correspond to the linear mixed-effects model regression. Significant correlations (*p* < 0.05) are formatted in bold.

*** *p* < 0.001.

## Discussion

### Season, Rather Than Warming, Dominates the Change of SOC

Regardless of season, warming had no significant effect on the SOC (Figure 1A). This result does not support hypothesis I (H1) and is consistent with the findings of other studies (Yue et al., 2015; Zhang et al., 2016; Jia et al., 2017; Guan et al., 2018; Jia et al., 2019; Wang et al., 2019; Chen et al., 2021) that indicated that warming did not dominate the change of SOC. Unlike the warming treatments, the content of SOC in August was significantly lower than that in May (Figure 1A), and the storage of SOC of August decreased by an average of 18.53 million grams of carbon per hectare (Figure 1I), indicating that alpine meadow soil is a carbon source rather than a carbon pool during the growing season. This result supports hypothesis 2 (H2) and is similar to that of another study in a grassland ecosystem (Yang et al., 2021), suggesting that the season, rather than warming, dominated the change in SOC (Gavazov et al., 2017). Since a study has confirmed that the soil carbon flow is higher than that in the non-growing season, we hypothesize that there is an “interannual cycle” process. In the “interannual cycle” process, the content of SOC changes from a “loss state” of the growing season to an “increase state” of the non-growing season and finally returns to the original level at the beginning of the growing season in the following year. In this study, warming reduced the aboveground biomass of plants, which in turn reduced the return of plant litter to the soil during the non-growing season. Therefore, warming did not cause the loss of SOC under the current experimental conditions, but it was expected that the alpine meadow SOC could be lost in future long-term climate warming.

Temperature is a key limiting factor for high-altitude ecosystems (Duan et al., 2019). In this study, the negative correlation between the soil temperature and SOC further confirmed that changes in soil temperature induced by warming or seasons could be used to explain the change in SOC (Supplementary Table S1). In particular, the maximum increase in temperature produced by the warming treatment in May and August was 2.25°C and 1.57°C, respectively. This is well below the increase in temperature caused by the season (5.14–5.82°C), which also indicated that the insignificant effect of warming on SOC could be because the increase in soil temperature caused by warming did not reach the temperature threshold of organic carbon change (Feng et al., 2022). Structural equation modeling also confirmed this (Figure 5). However, since the seasonal changes of SOC are likely to be a cumulative process, whether this inference is suitable for this study still merits further exploration.

### Warming Has Not Affected the Change of SOC During the Growing Season

The rate of change of SOC during the growing season was not significantly related to the rate of change of soil temperature (Table 2). This was inconsistent with the third hypothesis (H3) but confirms the conclusions of other studies (Classen et al., 2015; Yue et al., 2015), which found that the inter-seasonal temperature difference caused by the warming treatment did not impact microbiota, and thus, failed to alter the seasonal dynamic changes of SOC. However, this result is inconsistent with the results of some studies (Xu et al., 2018; Tian et al., 2021). Tian et al. (2021) and Xu et al. (2018) found that elevated soil temperatures indirectly affected the seasonal dynamics of the content of SOC by altering soil nutrition, physical protection, and microbial biomass. Plant biomass (Classen et al., 2015), soil respiration (Tian et al., 2021), and microbiota (Classen et al., 2015; Xu et al., 2018, 2021) are often used to explain the seasonal changes of SOC. In this study, the aboveground biomass gradually decreased with increasing temperature (Supplementary Figure S2A). Since the aboveground biomass of all the treatments was close to zero at the beginning of the growing season, the reduction in aboveground biomass caused by warming also illustrates the decrease in the seasonal difference of aboveground biomass under warming treatments. This also indicates that the seasonal difference of aboveground biomass had decreased as the temperature increased. Thus, we inferred that owing to the small input of plant aboveground carbon, the seasonal difference of SOC should be reduced as the temperature increases. However, this inference was inconsistent with the results of this study (Figure 1A), indicating that it was unreasonable to only use the plant aboveground biomass to predict the changes in SOC. Considering that belowground biomass and root exudates also play an important role in driving the change of SOC (Jansson and Hofmockel, 2020), further research needs to comprehensively consider belowground biomass and root exudates to explore the effect of warming on the seasonal changes of SOC. In addition to plant biomass, soil respiration is highly sensitive to temperature, and an increase in the temperature will activate soil respiration and accelerate its rate (García-Palacios et al., 2021). Since warming led to a gradual decrease in the seasonal temperature difference in this study (Supplementary Table S1), the seasonal difference of SOC will decrease as the temperature increases. However, we had not observed a decrease in the seasonal difference of SOC across a temperature gradient (Supplementary Figure S6), indicating that the seasonal change of SOC in this study cannot also be explained by the seasonal change of soil respiration. The insignificant correlation between the rate of change of soil respiration and that of the SOC effectively supported this (Table 2). Moreover, the decomposition of soil microbiota was another important reason for the seasonal change in SOC (Chen et al., 2015; García-Palacios et al., 2021; Tian et al., 2021). On the one hand, changes in the soil microbial community composition will affect the SOC by changing the activity of soil extracellular enzymes (Tian et al., 2021). In this study, warming did not have a significant effect on the seasonal differences of fungal and bacterial communities and the activities of soil extracellular enzymes. In addition, there was no significant correlation between the rate of change of the soil microbial community and the rate of change of the soil enzyme activity and that of SOC (Table 2). These results indicate that changes in soil microbial communities and soil enzyme activities in this study cannot explain the insignificant effect of warming on the seasonal differences of SOC. Alternatively, MBC, as an internal component of SOC, directly or indirectly affects the change in content of SOC (Table 2). In this study, the rate of change of MBC was significantly related to the rate of change of SOC. In addition, the SEM models also showed that warming failed to mediate seasonal changes in SOC by altering the changes in MBC (Figures 4B, 5B), indicating that the insignificant seasonal changes in soil temperature on microbial biomass caused by warming in this study could be an important reason for the insignificant effect of warming on the seasonal changes in SOC.

### MBC Was Highly Coupled to the Change of SOC

Although MBC comprises a small proportion of SOC (Figure 2D), both the random forest analysis and structural equation model all showed that MBC significantly correlated with the change in SOC, indicating a high degree of coupling of MBC to the change in SOC (Figures 4A, 5A). This is consistent with the findings of another study (Wang et al., 2021). This occurs because MBC, as an internal component of soil carbon (Mcdaniel et al., 2014; Ogle, 2018; Huang et al., 2021), has been regarded to be the liable carbon fraction of SOC that is closely related to the availability of soil nutrients (Guan et al., 2018) and indirectly participates in the key process of the soil carbon cycle (Ma et al., 2020). In addition, our results had shown that neither season nor warming had a significant effect on the ratio of MBC/MBN. This was also confirmed by another study on an alpine meadow ecosystem on the Qinghai–Tibet Plateau, in which the MBC/MBN remained stable under the warming treatment (Wang et al., 2014). However, this result contradicts another study in a subtropical ecosystem that confirmed that warming produces positive effects on MBC/MBN by increasing the decomposition of soil organic matter (Gao et al., 2017). This inconsistency is primarily determined by the climate characteristics of the two types of ecosystems. In particular, the characteristic cold climates of alpine meadows do not facilitate the decomposition of organic matter, but the opposite is true for subtropical climates. In addition to the decomposition of organic matter, the microbial community dominated by fungi (or bacteria) should not be confirmed to be associated with a high (or low) ratio of MBC/MBN (Gao et al., 2021). However, the ratio of l g ITS/l g 16S rRNA gene copy numbers was always approximately equal to 1 under all the treatments, suggesting a co-dominance of fungi and bacteria. This also explained the stability of MBC/MBN ratio under every treatment in this study. However, what was anomalous was that although the MBC/MBN ratio changed owing to warming or season, it was shown to be another variable that was closely associated with the rate of change of SOC. However, both the random forest analysis and the structural equation model also showed that the rate of change of the content of SOC was determined by the rate of change of MBC rather than MBN (Figure 5). Therefore, we inferred that the contribution of MBC/MBN to the change in SOC is just another manifestation of the contribution of MBC and is not really driven by the ratio of MBC/MBN, indicating that the microbiological properties of soil that were related to the carbon cycle were “carbon-related” rather than “nitrogen-related.”

In addition, warming had no significant effect on the content of DOC regardless of season (Figure 1D). This result contradicts those of other studies (Six et al., 2004; Li et al., 2016; Tian et al., 2021). One of these studies found that warming can increase the content of DOC by increasing the dissolved organic compounds produced by plants or microbiota (Bai et al., 2020). However, other studies have found that warming can reduce the organic carbon content dissolved in the soil by promoting the formation of aggregates to solidify the DOC (Six et al., 2004; Li et al., 2016; Tian et al., 2021). In addition, the rate of change of DOC can mediate the rate of change of the SOC by changing the rate of change of MBC during the growing season (Figure 5B). These results are consistent with those of other studies (Zhang et al., 2015; Xiong et al., 2016; Bhople et al., 2019; Deltedesco et al., 2020) that further confirm that the DOC participates in the process of regulation of soil carbon cycle by affecting MBC-derived carbon as described in the carbon cycle model by Fan et al. (2021). However, since the content of DOC makes only minor contributions to the variation in the content of SOC (Chen et al., 2015), its impact on SOC stability was likely to be achieved only through its impact on MBC. Integrating this analysis, MBC, the ratio of MBC/MBN, and DOC are all variables that are significantly associated with the change in the content of SOC, but the effects of both MBC/MBN and DOC are essentially mediated by MBC. There was a high degree of coupling between MBC and soil organic carbon. In addition, MBC is more sensitive to temperature than SOC (Walker et al., 2018). Thus, strengthening the research on the temperature sensitivity of MBC can improve our ability to predict changes in SOC under climate warming.

## Conclusion

**Figure 6:**
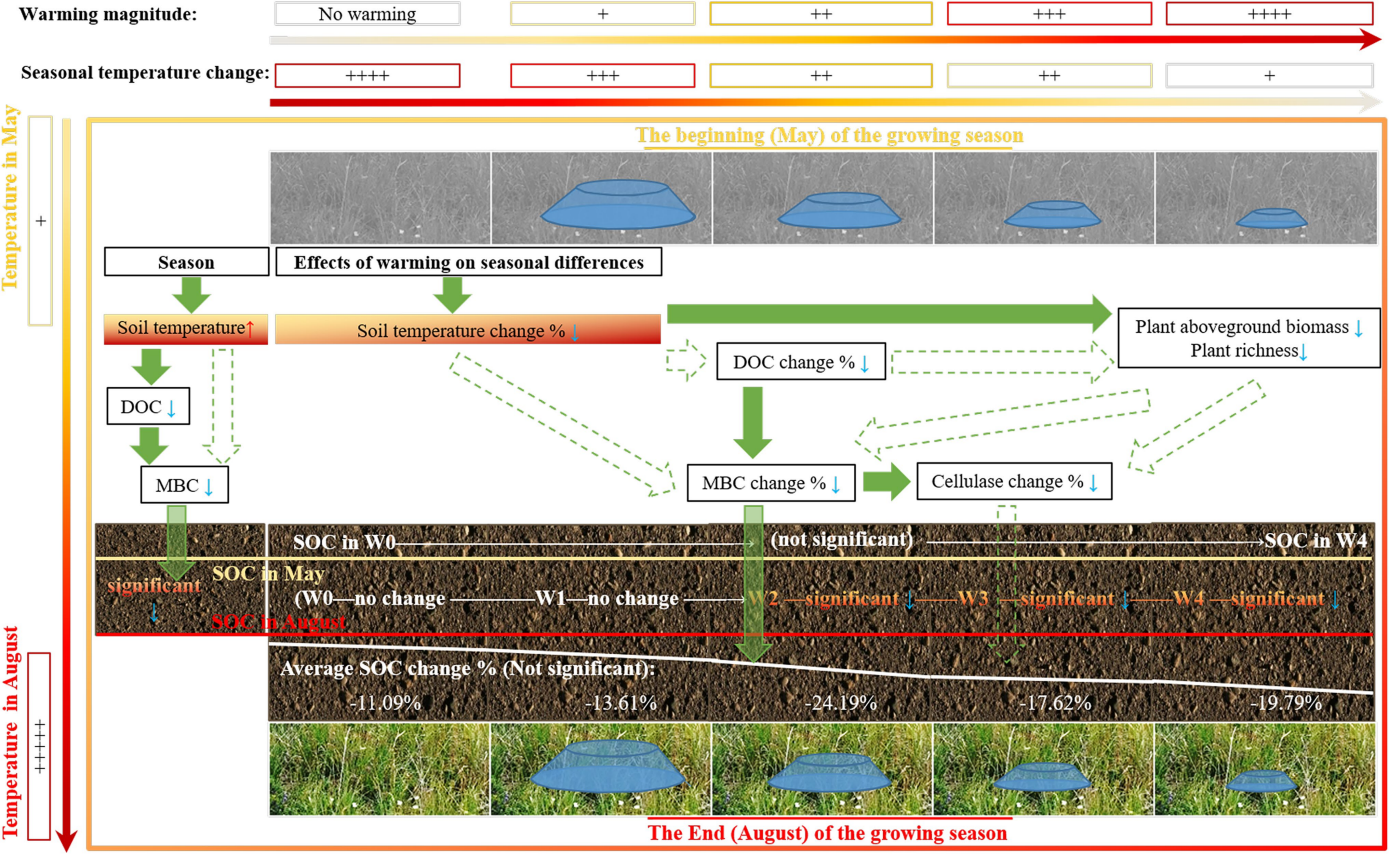
Schematic representation of the effect of experimental warming on the change of soil organic carbon during the growing season.

This study found that SOC at the end of the growing season was lower than that at the beginning of the growing season, and this seasonal change of SOC during the growing season remained unchanged following warming treatments (Figure 6). These findings indicate that alpine meadow soil was a source of carbon during the growing season, but climate warming had no significant impact on it. Our findings have important implications for improving our understanding of the effects of climate warming on the change in SOC during the growing season, suggesting that in the regulation of carbon source or pool in alpine meadow ecosystem, more attention should be paid to changes in SOC during the growing season, rather than climate warming.

## Data Availability Statement

The datasets presented in this study can be found in online repositories. The names of the repository/repositories and accession number(s) can be found at: https://www.ncbi.nlm.nih.gov/, PRJNA719774; https://www.ncbi.nlm.nih.gov/, PRJNA719776.

## Author Contributions

GS, YL, HZ, and HF designed the study. GS, LM, ZZ, and SJ performed field work and done experiments with the help of YY and JP. YY carried out statistical and bioinformatics analysis, prepared figures and tables, and done results interpretations and wrote the manuscript with help from GS and YL. GS, YL, QZ, BY, HZ, and HF reviewed and edited the manuscript. GS, YL, HZ, and HF acquired the funds for the study. All authors contributed to the article and approved the submitted version.

## Funding

This research was funded financially by the Second Tibetan Plateau Scientific Expedition and Research (STEP) program (Nos. 2019QZKK0301 and 2019QZKK0302), the National Natural Science Foundation of China (Nos. 31860146; 31971445; 31870494; 32171579 and U21A20186), the Outstanding Youth Foundation of Gansu Province (No. 20JR5RA500), the CAS “Light of West China” Program, “Qinghai Provincial Natural Science Foundation Innovation Team Project (2021-ZJ-902),” and the Joint Research Project of Three-River-Resource National Park (No. LHZX-2020-08).

## Conflict of Interest

The authors declare that the research was conducted in the absence of any commercial or financial relationships that could be construed as a potential conflict of interest.

## Publisher’s Note

All claims expressed in this article are solely those of the authors and do not necessarily represent those of their affiliated organizations, or those of the publisher, the editors and the reviewers. Any product that may be evaluated in this article, or claim that may be made by its manufacturer, is not guaranteed or endorsed by the publisher.

## Footnotes

^1^ https://unite.ut.ee/

^2^ http://www.arb-silva.de

^3^ http://www.r-project.org/

^4^ https://github.com/microbiota/amplicon

^5^ http://huttenhower.sph.harvard.edu/galaxy/
