# Supplementary material for: Experimental Warming Has Not Affected the Changes in Soil Organic Carbon During the Growing Season in an Alpine Meadow Ecosystem on the Qinghai–Tibet Plateau
Source: Front Plant Sci. 2022 Mar 18;13:847680. doi: 10.3389/fpls.2022.847680 (PMC8971846; doi:10.3389/fpls.2022.847680)
Supplement: Supplementary file 1 [file Data_Sheet_1.docx]

Supplementary Material

**Table S1** Temperatures in the air and soil for the annual growing season in the different treatments. ΔT represents changes in the soil or air temperature in the different treatments. Air_10_ and Air_20_ represent the air temperatures at 10 cm and 20 cm aboveground, respectively.

|  | Season | W0 | W1 | W2 | W3 | W4 |
| --- | --- | --- | --- | --- | --- | --- |
| Air_20_ (℃) | May | 6.44 | 7.24 | 7.4 | 7.54 | 8.42 |
|  | August | 11.06 | 11.16 | 11.51 | 11.69 | 11.88 |
| ΔT_air20_ | | 4.62 | 3.92 | 4.11 | 4.15 | 3.46 |
| Air_10_ (℃) | May | 6.11 | 6.56 | 6.82 | 7.05 | 7.54 |
|  | August | 13.54 | 14.02 | 14.11 | 14.38 | 14.49 |
| ΔT_air10_ | | 7.43 | 7.46 | 7.29 | 7.33 | 6.95 |
| Soil temperature (℃) | May | 6.64 | 7.37 | 8.24 | 8.37 | 8.89 |
|  | August | 12.46 | 12.98 | 13.43 | 13.95 | 14.03 |
| ΔT_soil_ | | 5.82 | 5.61 | 5.19 | 5.58 | 5.14 |

**Table S2** Summary of high-throughput sequencing.

|  | **Fungi** | **Bacteria** |
| --- | --- | --- |
| **Summary of the sequencing statistics:** |  |  |
| Amplified region | ITS1F-ITS2R | 338F-806R |
| Insert size | 300 | 468 |
| Sequencing length | PE300 | PE300 |
| Total raw reads | 3475505*2 | 2581054*2 |
| Total raw base (bp) | 2092254010 | 1553794508 |
| Clean min length | 50 | 50 |
| Total clean base (bp) | 770833845 | 1079854247 |
| Average clean reads length | 221.7904578 | 418.3772393 |
| Total representative OTUs | 3,181 | 8,724 |
| Clean representative OTUs (for alpha diversity) | 2,569 | 7,876 |
| Filtered representative OTUs (for beta diversity, after rarefaction, total abundance>0) | 1,935 | 5,337 |
| Normalization reads per sample (for beta diversity) | 47,600 | 25,025 |
| **Summary of the targeted taxonomy ranks (unclassified taxonomy included):** |  |  |
| Domain | 1 | 1 |
| Kingdom | 1 | No rank |
| Phylum | 13 | 36 |
| Class | 36 | 81 |
| Order | 74 | 180 |
| Family | 147 | 239 |
| Genus | 307 | 407 |

OTUs, operational taxonomic units.

**Figure S1** The field experimental system of warming and winter grazing at the Haibei Experimental Station, Chinese Academy of Sciences. A total of 25 plots composed of five temperature treatments (ambient temperature and four warming treatments) with five repetitions were distributed into subplots that were divided into five rows and five columns based on a random block design. Four levels of warming treatments were achieved by installing four types of conical OTCs constructed of 1.0 mm thick fiberglass. The four types of OTCs were 40 cm high, but the top and bottom diameters were 1.6 m and 2.05 m, respectively (W1), 1.3 m and 1.75 m, respectively (W2), 1.0 m and 1.45 m, respectively (W3), and 0.7 m and 1.15 m (W4), respectively. OTCs, open top chambers.

**Figure S2** Aboveground biomass (A), species richness (B) and the results of principal coordinate analysis (PCoA) (C) of plant communities.

**Figure S3** Rarefaction curves of the number of OTUs for fungal (A) and bacterial (B) communities in different treatments. OTUs, operational taxonomic units.

**Figure S4** Relative abundance of the top 10 phyla of the fungal and bacterial communities.

**Figure S5** The results of LEfSe analyses for differences in the relative abundance of OTUs in fungal (A) and bacterial communities (B) between May and August. The LEfSe analyses were conducted using linear discriminant analysis (LDA). Clades and nodes with significant differences (LDA score > 2.0) are shown in cladograms. May and August are represented in red and green, respectively. LEfSe, linear discriminant Effect Size; OTUs, operational taxonomic units.

**Figure S6** The rate of change of soil and microbial properties during the growing season in the different treatments. **p* < 0.05. ***p* < 0.01. ****p* < 0.001.
